# Genetic analysis of 37 cases with primary periodic paralysis in Chinese patients

**DOI:** 10.1186/s13023-024-03170-5

**Published:** 2024-04-12

**Authors:** Xuechao Zhao, Haofeng Ning, Lina Liu, Chaofeng Zhu, Yinghui Zhang, Guifang Sun, Huanan Ren, Xiangdong Kong

**Affiliations:** 1 The Genetics and Prenatal Diagnosis Center, The Department of Obstetrics and Gynecology, The First Affiliated Hospital of Zhengzhou University, Jianshe Rd, Erqi District, 450052 Zhengzhou, Henan China; 2 Obstetrics and Gynaecology, The Seventh Affiliated Hospital, Sun Yat-Sen University, No 628 Zhenyuan Road Guangming District, 518107 Shenzhen, PR China; 3 The Department of Endocrinology, The First Affiliated Hospital of Zhengzhou University, Jianshe Rd, Erqi District, 450052 Zhengzhou, Henan China; 4 The Department of Neurology, The First Affiliated Hospital of Zhengzhou University, Jianshe Rd, Erqi District, 450052 Zhengzhou, Henan China

**Keywords:** Primary periodic paralysis, Hypokalemic periodic paralysis, *CACNA1S* and *SCN4A* genes, Panel and WES, Minigene

## Abstract

**Background:**

Primary periodic paralysis (PPP) is an inherited disorders of ion channel dysfunction characterized by recurrent episodes of flaccid muscle weakness, which can classified as hypokalemic (HypoPP), normokalemic (NormoPP), or hyperkalemic (HyperPP) according to the potassium level during the paralytic attacks. However, PPP is charactered by remarkable clinical and genetic heterogeneity, and the diagnosis of suspected patients is based on the characteristic clinical presentation then confirmed by genetic testing. At present, there are only limited cohort studies on PPP in the Chinese population.

**Results:**

We included 37 patients with a clinical diagnosis of PPP. Eleven (29.7%) patients were tested using a specific gene panel and 26 (70.3%) by the whole-exome sequencing (WES). Twenty-two cases had a genetic variant identified, representing a diagnostic rate of 59.5% (22/37). All the identified mutations were either in the *SCN4A* or the *CACNA1S* gene. The overall detection rate was comparable between the panel (54.5%: 6/11) and WES (61.5%: 16/26). The remaining patients unresolved through panel sequencing were further analyzed by WES, without the detection of any mutation. The novel atypical splicing variant c.2020-5G > A affects the normal splicing of the *SCN4A* mRNA, which was confirmed by minigene splicing assay. Among 21 patients with HypoPP, 15 patients were classified as HypoPP-2 with *SCN4A* variants, and 6 HypoPP-1 patients had *CACNA1S* variants.

**Conclusions:**

Our results suggest that *SCN4A* alleles are the main cause in our cohort, with the remainder caused by *CACNA1S* alleles, which are the predominant cause in Europe and the United States. Additionally, this study identified 3 novel *SCN4A* and 2 novel *CACNA1S* variants, broadening the mutation spectrum of genes associated with PPP.

**Supplementary Information:**

The online version contains supplementary material available at 10.1186/s13023-024-03170-5.

## Background

Primary periodic paralysis (PPP) is an inherited disorder of ion channel dysfunction characterized by recurrent episodes of flaccid muscle weakness [1]. The attacks may last from hours to days or weeks, patients usually completely recovery between the attacks. PPP is respectively classified as HypoPP, NormoPP or HyperPP according to low, normal, or elevated concentration potassium levels during the paralytic attacks [2, 3]. Attacks of weakness may be precipitated by a high carbohydrate diet, resting post exercise, environmental cold or heat, as well as poor sleep. Additional triggers can include stress/excitement/fear, salt intake, prolonged immobility and the use of glucosteroids or alcohol [4–6]. During episodes of weakness, arms or legs may be preferentially affected. The attack frequency is highly variable (ranging from 1 to 2 to a dozen times a month), and the duration of episodes of weakness is also variable (several hours to days).

PPP is attributed to mutations in genes encoding subunits of channel proteins in the skeletal muscle membrane or endoplasmic reticulum like sodium, potassium, and calcium channels [7]. The most commonly affected genes include those encoding the alpha subunit of the skeletal muscle L-type Cav 1.1 channel (*CACNA1S*: OMIM: *603,967), the skeletal muscle Nav1.4 channel (*SCN4A*: OMIM: *114,208) and potassium channels Kir2.1, Kir3.4 (*KCNJ2*, *KCNJ5*) [8]. HypoPP is often caused by mutations in *CACNA1S* (HypoPP-1) or *SCN4A* (HypoPP-2) [9]. The clinical presentation is identical for patients with HypoPP-1 and HypoPP-2, because gene defects of either channel cause an abnormal leakage current, which is active at the resting potential and produces susceptibility to paradoxical depolarization of the muscle fibers and inexcitability at low extracellular K^+^ concentrations [6, 10]. For patients suspected of periodic paralysis, diagnosis is based on the characteristic clinical presentation then confirmed by genetic testing, which identifies a heterozygous pathogenic mutation in 60–70% of patients meeting clinical criteria [11, 12].

Prior to the development of next-generation sequencing (NGS), the genetic diagnosis of PPP was based on hot spots or exon-by-exon screening of the reported genes by Sanger sequencing, a time-consuming method [9]. The targeted sequencing (panel) is considered as a cost-effective strategy for the genetic diagnosis of PPP during the past years [13]. With the continuous development of NGS technology and cost reduction in recent years, WES has also become a feasible strategy for the genetic analysis of PPP cases with more complicated or unusual etiologies.

HypoPP is the most frequent form of periodic paralysis in humans, with a prevalence rate of approximately 1 in 100,000 live births [2].About 60% of patients are classified as HypoPP-1 because they carry variants of the *CACNA1S* gene; while 20% patients are classified as HypoPP-2 with *SCN4A* gene variants [2]. However, the reported cohort studies on PPP mainly focused on patients from the United States and Europe, and there are limited cohort studies on PPP in the Chinese population. In this study, we reviewed the clinical manifestations and analyzed the genetic characteristics of 37 patients with PPP from China by targeted panel sequencing and WES.

## Materials and methods

### Patients and inclusion criteria

The patients were referred to our center for genetic testing by specialists from the neurology and endocrinology departments of our hospital. The recruitment of patients with clinically defined PPP was based on the following criteria: (1) The main clinical manifestations were episodic flaccid weakness, with or without abnormal serum K^+^; the details included “history of at least two attacks of flaccid limb weakness (including weakness of the muscles of the eyes, throat, breathing muscles, or trunk)” or “one attack of muscle weakness in the proband and one attack of weakness in one relative”; (2) “duration of attack (muscle weakness involving ≥ 1 limbs) ranging from minutes to days; (3) normal psychomotor development. Patients were excluded from secondary periodic paralysis, such as hyperthyroidism, renal tubular acidosis, or primary aldosteronism etc based on blood-gas analysis, aldosterone and renin-angiotensin levels, thyroid function, ultrasound analysis of the thyroid and adrenal glands. Based on these criteria, we included a total of 37 patients from unrelated Chinese families for further genetic analysis.

### Samples and DNA extraction

Genomic DNA was extracted from EDTA -stabilized peripheral blood samples using the Lab-Aid® 824 DNA Extraction Kit (ZEESAN, Xiamen, China), according to the manufacturer’s protocol.

### Ion torrent PGM sequencing

For patients 4,7–9,17 and 19, who were enrolled from January 2019 to July 2020, a specific gene panel analysis was performed. This panel included the whole *CACNA1S* and *SCN4A* genes, with 5 additional genes related to ion channelopathies, including *RYR1, KCNE3, KCNJ18, KCNJ2* and *KCNJ5*. The library preparation was performed by amplifying 10 ng of genomic DNA, using the Ion AmpliSeq™ Library Kit 2.0 (Life Technologies), resulting in a barcoded library of the 178 exons of the *CACNA1S/SCN4A/RYR1/KCNE3/KCNJ18/KCNJ2/KCNJ5* genes compatible with the Ion PGM platform, according to the Life Technologies protocol. Libraries were purified using the Agencourt® AMPure® XP system, quantified using the Qubit® dsDNA HS Assay Kit (Invitrogen Corporation, Life Technologies, Carlsbad, CA), pooled at an equimolar ratio, annealed to carrier spheres (Ion Sphere™ Particles, Life Technologies) and clonally amplified by emulsion PCR (emPCR) using the Ion OneTouch™ 2 system (Ion PGM™ Template Hi-Q™ view OT2 200 kit, Life Technologies). The spheres, carrying single-stranded DNA templates, were loaded to 316™v2 chip and sequenced on the Ion Torrent PGM, using the Ion PGM™ Hi-Q™ view Sequencing 200 kit v2, according to the protocol of Life Technologies. Post-run analysis was conducted using the latest version (v5.10) of the data analysis software Torrent Suite™ (Life Technologies). Coverage assessment was performed using the “coverage Analysis” plug-in (v5.10) that gives information about the amplicons read coverage and variants were called using the “variant Caller” plug-in (5.10). Detailed experimental procedures have already been reported previously [14].

### Sequence analysis and variant annotation of WES

For patients 1–3, 5–6, 10, 11–16, 18 and 20–22, enrolled from August 2020 to December 2022, WES was performed. Genomic DNA was quantified using Qubit 4.0 (Thermo Fisher Scientific Inc. USA). DNA libraries were established using the Ada & Index Kit (UDI for LIM) and Enzyme Plus Library Prep Kit (iGeneTech Co., Ltd, Beijing, China), while exome coding and splicing regions were captured using the AIExome Human Exome Panel V2 Plus with TargetSeq One Hyb & Wash Kit (iGeneTech Co., Ltd, Beijing, China), according to the manufacturer’s instructions. Subsequently, the captured libraries were sequenced on the NovaSeq6000 platform (Illumina, San Diego, CA, USA) with an average sequencing depth > 100× and 20× sequencing coverage > 98%. Sentieon (release 201,808.05) was used to align paired-end reads to the human reference genome GRCh37/ hg19 as stipulated by the Genome Analysis Toolkit(GATK) best practice guidelines, and the duplicate reads were removed using Picard version 2.9.0 [15]. All variants were annotated using databases including the 1000 genomes project, dbSNP, the genome aggregation database, ClinVar, the Human Genomic Mutation Database and OMIM. All candidate gene variants were confirmed by Sanger sequencing to validate the genotype of the proband, parents, and other members if there was a family history of PPP. Variants were classified into the five categories -“pathogenic”, “likely pathogenic ”, “uncertain significance”, “likely benign” and “benign”-according to the American College of Medical Genetics and Genomics (ACMG) guidelines for interpretation of genetic variants [16].

### Cell culture and transfection

HeLa and HEK293T cells were maintained in Dulbecco’s modified Eagle’s medium (DMEM) with 10% FBS, penicillin (50 U/mL), and streptomycin (50 mg/mL) at 37 ℃ in a humidified incubator with 5% CO_2_. Cells were transfected with the SCN4A-pcMINI-wt/mut plasmids using 1 mg/mL polyethyleneimine (PEI; Sigma, St. Louis, MO). Briefly, 1.0 × 10^5^ 293T cells or 6 × 10^4^ HeLa cells were seeded into each well of a 12-well plate, and cultured in DMEM containing 10% FBS for 20 h before transfection. Approximately 1 µg of plasmid DNA was suspended in 100 µL of DMEM in a polystyrene tube, after which PEI (3 µL for 293T or 6 µL for HeLa) was added and immediately mixed before incubation for 10 min at room temperature. Thereafter, the mixture was gently dispensed into the wells and incubated at 37 ℃ in a humidified incubator with 5% CO_2_. The medium was exchanged with complete medium (DMEM supplemented with FBS and antibiotics) 8 h later [17].

### Minigene splicing assay

We predicted the effect of the mutation site c.2020-5G > A on mRNA splicing using the online tool SpliceAI (https://spliceailookup.broadinstitute.org/). The results suggested that the variant generates a new acceptor site (score = 0.98). Activation of a cryptic acceptor site results in the retention of 3 bp of Intron12 in the cDNA.

For the minigene splicing assay, exon 13 of *SCN4A* and flanking intron sequences (Intron12 (481 bp)-Exon13 (357 bp)-part Intron13 (233 bp)) was amplified from the genomic DNA of the patient using the primers: SCN4A-pcMINI-KpnI-F (ggtaGGTACCggagttttgttctgtagccc) and SCN4A-pcMINI-EcoRI-R (tgcaGAATTCctaggcccagagctgctgaa). After treatment with restriction endonucleases *Kpn* I and *EcoR* I, the amplified fragments were cloned into the splicing reporter plasmid pcMINI, containing the sequence ExonA-IntronA-MCS-IntronB-ExonB (Bioeagle, China) to construct SCN4A-pcMINI-wt/mut as described previously [18]. Wild-type and mutant recombinants were selected by monoclonal sequencing. Total RNA was isolated from HeLa and 293T cells transiently transfected with the WT or mutant plasmids using TRIzol™ Reagent ( Invitrogen™, USA), according to the manufacturer’s instructions. Samples were analyzed by RT-PCR using SuperScript IV® Reverse Transcriptase (Invitrogen) using the primers: pcMINI-F (CTAGAGAACCCACTGCTTAC) and pcMINI-R (TAGAAGGCACAG TCGAGG) followed by 1% agarose gel electrophoresis. We next ligated the PCR product into the T vector and picked out individual colonies for sequencing. The normal-expected size includes the Exon A-Exon13-Exon B fragment with a size of 746 bp between the primers pcMINI-F and pcMINI-R.

## Results

### Clinical features

Among the 22 probands, 21 were classified as HypoPP and 1 HyperPP according to the K^+^ levels during the paralytic attacks. The mean age of probands when collecting the blood samples was 24.5 ± 12.2 years, and the mean onset age was 19.7 ± 11.0 years. The gender ratio was heavily skewed, with 95.4% males (21/22) and 4.6% females (1/22). Triggers for first attack include a high carbohydrate diet, resting post exercise, environmental cold or heat, and poor sleep. Muscle weakness often involved the two legs and four limbs, while the duration of attacks ranged from minutes to days. A brief clinical summary of the patients is shown in Table 1.

**Table 1:** Clinical data of patients with primary periodic paralysis

Patients No.	Sex	Age(y)	Onset age(y)	Preciptating factors for first-onset	Myasthenia	Duraion	Frequency (Times/year)	Phenotype
1	M	31	25	High carbohydrate diet	Two legs, paroxysmal	Several hours to days	5–8	HypoPP
2	M	14	13	resting post exercise	Two legs, paroxysmal	Several days	9	HypoPP
3	M	13	12.5	High carbohydrate diet	Four limbs, paroxysmal	Several days	4	HypoPP
4	M	21	18	heat	Two legs, paroxysmal	Several days	10–20	HypoPP
5	M	3	1	environmental cold	Two legs, paroxysmal	Several hours	6–8	HyperPP
6	M	43	23	resting post exercise	Four limbs, paroxysmal	Several days	8–12	HypoPP
7	M	33	21	High carbohydrate diet	Four limbs, paroxysmal	Several days	7–11	HypoPP
8	M	17	13	resting post exercise	Four limbs, paroxysmal	Several days	4–8	HypoPP
9	M	18	15	High carbohydrate diet	Four limbs, paroxysmal	Several days	3–8	HypoPP
10	M	27	15	Poor sleep	Four limbs, paroxysmal	Several days	5–8	HypoPP
11	M	34	33.5	high carbohydrate diet, resting post exercise	Four limbs, paroxysmal	Several days	3	HypoPP
12	M	24	18	heat	Four limbs, paroxysmal	Several hours to days	5–7	HypoPP
13	M	26	23	heat	Four limbs, paroxysmal, diabetes	Several days	4–7	HypoPP
14	M	17	13	resting post exercise	Four limbs, paroxysmal	Several days	6–8	HypoPP
15	M	17	15	High carbohydrate diet	Two legs, paroxysmal	Several days	7–10	HypoPP
16	M	13	12	heat	Two legs, paroxysmal	Several days	9–12	HypoPP
17	F	26	16	resting post exercise	Four limbs, paroxysmal	Several days	6–10	HypoPP
18	M	41	35	resting post exercise	Four limbs, paroxysmal	Several days	7–9	HypoPP
19	M	18	15	resting post exercise	Four limbs, paroxysmal	Several days	4–8	HypoPP
20	M	18	16	heat	Four limbs, paroxysmal	Several days	3–5	HypoPP
21	M	28	24	heat	Two legs, paroxysmal	Several days	1–2	HypoPP
22	M	58	56	resting post exercise	Two legs, paroxysmal	Several days	4–6	HypoPP

### Genetic analysis

**Fig. 1:**
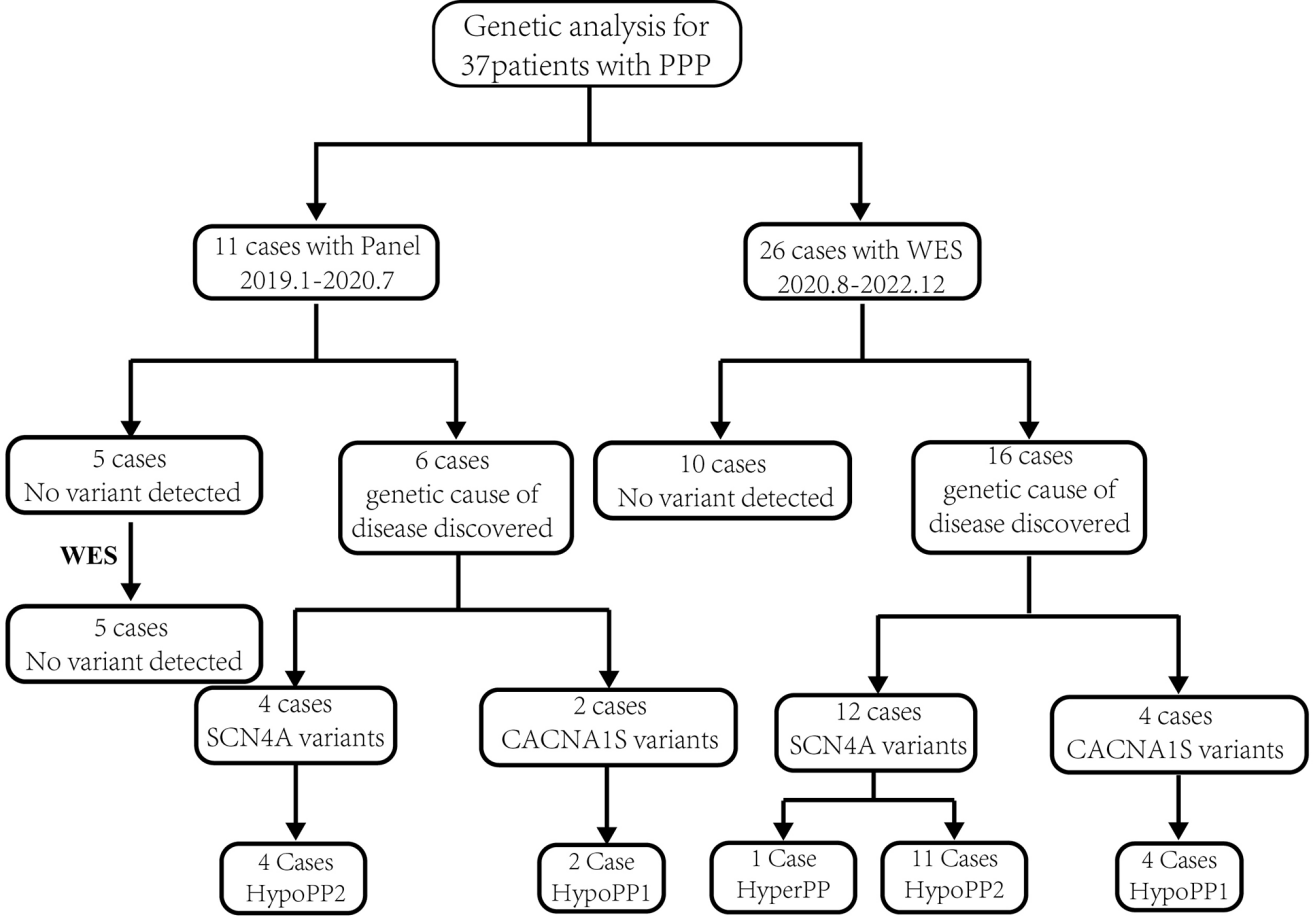
Flowchart showing the methods and results of 37 cases enrolled in our study. Panel: specific gene panel analysis; WES: whole-exome sequencing

A total of 37 patients with suspected PPP were studied, 11 of which (29.7%) were investigated using a specific gene panel and 26 (70.3%) using the WES. Overall, 22 cases had a genetic variant identified, corresponding to a diagnostic rate of 59.5% (22/37). All patients had mutations in either *SCN4A* or *CACNA1S*, including 3 unreported *SCN4A* variants and 2 unreported *CACNA1S* variants. Mutations were identified in 6 out of 11 probands (54.5%) using the panel, as well as 16 out of 26 probands (61.5%) using WES. The remaining patients unresolved using the panel were further analyzed using WES, without the detection of any mutations (Fig. 1). Mutations in *SCN4A* were detected in 16 out of the 22 (72.7%) genetically defined patients and *CACNA1S* mutations in 6 (27.3%) (Table 2). The most frequent mutation was c.2024G > A (p.Arg675Gln) in the *SCN4A* gene, found in 4 HypoPP patients. Two out of 6 *CACNA1S* mutations were c.3716G > A (p.Arg1239His) in two unrelated families.

**Table 2:** The gene mutation information of patients with primary periodic paralysis

Patients No.	Gene	Mutation	Exon	Structural position	reported	Variant classification (ACMG)	Variant source	Family history	Methods
1	*SCN4A*	c.2024G > A (p.Arg675Gln)	13	DII-S4	reported [45]	P(PS1 + PS3 + PM1 + PM2 + PP3)	Father	-	WES
2	*SCN4A*	c.2024G > A (p.Arg675Gln)	13	DII-S4	reported [45]	P(PS1 + PS3 + PM1 + PM2 + PP3)	Mother	+	WES
3	*SCN4A*	c.2024G > A (p.Arg675Gln)	13	DII-S4	reported [45]	P(PS1 + PS3 + PM1 + PM2 + PP3)	Father	-	WES
4	*SCN4A*	c.2024G > A (p.Arg675Gln)	13	DII-S4	reported [45]	P(PS1 + PS3 + PM1 + PM2 + PP3)	Mother	-	Panel
5	*SCN4A*	c.2111 C > T (p.Thr704Met)	13	DII-S5	reported [50]	P(PS4 + PM1 + PM2 + PP3)	Father	+	WES
6	*SCN4A*	c.2143G > A (p.Ala715Thr)	13	DII-S5	reported [38]	LP(PM1 + PM2 + PM5 + PP3)	UN	-	WES
7	*SCN4A*	c.4774 A > G (p.Met1592Val)	24	DIV-S6	reported [39]	P(PS1 + PS3 + PM1 + PM2 + PP3)	Father	+	Panel
8	*SCN4A*	c.2015G > A (p.Arg672His)	12	DII-S4	reported [27]	P(PS1 + PS3 + PM1 + PM2 + PP3)	Mother	+	Panel
9	*SCN4A*	c.664 C > T (p.Arg222Trp)	5	DI-S4	reported [12]	P(PS1 + PS3 + PM1 + PM2 + PP3)	Mother	-	Panel
10	*SCN4A*	c.4183 A > G (p.Ile1395Val)	23	DIV-S2	unreported	VUS(PM1 + PM2)	UN	-	WES
11	*SCN4A*	c.1414 C > A (p.Leu472Ile)	9	Cyto	unreported	VUS(PM2)	UN	-	WES
12	*SCN4A*	c.2020-5G > A (p.Leu673_Leu674insGln)	13	DII-S4	unreported	LP(PS4 + PM2 + PP3)	Father	+	WES
13	*SCN4A*	c.2020-5G > A (p.Leu673_Leu674insGln)	13	DII-S4	unreported	LP(PS4 + PM2 + PP3)	UN	-	WES
14	*SCN4A*	c.2014 C > T(Arg672Cys)	12	DII-S4	reported [36]	LP(PM1 + PM2 + PP3 + PP4)	Mother	+	WES
15	*SCN4A*	c.1354G > A(p.Glu452Lys)	9	Cyto	reported [40]	LP(PM1 + PM2 + PP3 + PP4)	UN	-	WES
16	*SCN4A*	c.3404G > A(p.R1135H)	18	DIII-S4	reported [12]	P(PM1 + PM2 + PS4_S + PS3_S + PP3 + PP4)	Father	+	WES
17	*CACNA1S*	c.3716G > A (p.Arg1239His)	30	DIV-S4	reported [29]	P(PS4 + PM1 + PM2 + PP5 + PP3)	Mother	+	Panel
18	*CACNA1S*	c.3716G > A (p.Arg1239His)	30	DIV-S4	reported [29]	P(PS4 + PM1 + PM2 + PP5 + PP3)	UN	-	WES
19	*CACNA1S*	c.1408G > A(p.V470M)	11	DII-S2	reported [41]	VUS(PM2 + PP3)	Mother	-	panel
20	*CACNA1S*	c.1582 C > T(p.R528C)	11	DII-S4	reported [30]	LP(PM1 + PM2 + PM5 + PP3 + PP1)	Father	+	WES
21	*CACNA1S*	c.704 C > T(p.Ala235Val)	6	EX(extracellular)	unreported	LP(PM1 + PM2 + PP3 + PP4)	UN	-	WES
22	*CACNA1S*	c.3905G > A (p.Arg1302Gln)	32	EX(extracellular)	unreported	VUS(PM1 + PM2 + PP3)	UN	-	WES

RefSeq numbers for *SCN4A*: NM_000334; *CACNA1S*: NM_000069. P: pathogenic; LP: likely pathogenic; VUS: uncertain significance; UN: Unknown

**Fig. 2:**
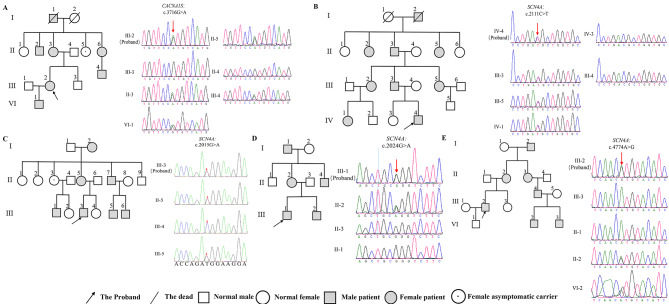
Pedigree and genetic analysis of five patients with primary periodic paralysis. **(A)** Patient 17, *CACNA1S* c.3716G > A mutation. **(B)** Patient 5, *SCN4A* c.2111 C > T (p.T704M) mutation. **(C)** Patient 8, *SCN4A* c.2015G > A (p.R675Q) mutation. **(D)** Patient 2, *SCN4A* c.2024G > A mutation **(E)** Patient 7, *SCN4A* c.4774 A > G mutation

A total of 9 cases had a family history of periodic paralysis. Five large pedigrees contained multiple patients over 3 generations (5 unrelated patients plus 30 of their affected relatives). Among them, 3 families with HypoPP and 1 family with HyperPP carried mutations in the *SCN4A* gene (31 patients), while 1 carried a mutation in the *CACNA1S* gene (7 patients) (Fig. 2).

### Case examples

#### Case 12

The proband was a 24-year-old male who presented with for “paroxysmal limb weakness for 6 years”. The patient developed weakness in the limbs after having a cold and fever 6 years ago. Electrolyte examination at the local hospital indicated hypokalemia. After intravenous potassium supplementation, the symptoms were relieved significantly. During 6 years, the symptoms occurred intermittently in the morning. During the attacks, the blood potassium fluctuated at 2.0–3.0 mmol/L (reference interval, 3.5–5.5 mmol/L). Six days before referral to our hospital, the above symptoms occurred again, and he went to the local hospital for blood potassium examination, which revealed a value of 2.22 mmol/L. After receiving potassium supplementation treatment, the symptoms were relieved and blood potassium increased to 4.0 mmol/L, dropping to 2.99 mmol/L again after 4 days. WES analysis confirmed that case 12 was heterozygous for the *SCN4A* c.2020-5G > A variant, inherited from his father, who also had a history of hypokalemic episodes.

#### Case 13

The proband was a 26-year-old male referred to our hospital due to “pain and weakness in all limbs for 13 days”. He had been hospitalized for periodic paralysis 3 years prior and had a history of diabetes for 2 years. After a cold infection, he developed pain and weakness in both lower limbs and could walk. Limb pain was mainly proximal, and the weakness symptoms gradually aggravated. Ten days before presentation at our hospital, he was weak, unable to walk, and fell to the ground. The symptoms slightly decreased after two hours and improved after potassium supplementation. The limb pain and weakness worsened again during the 3 most recent days. The blood potassium was 2.78 mmol/L during the attack. There was no family history of any disease similar to the patient. A heterozygous c.2020-5G > A variant of the *SCN4A* gene was confirmed by WES.

### Splicing study of the c.2020-5G > A variant of ***SCN4A*** using the minigene assay

**Fig. 3:**
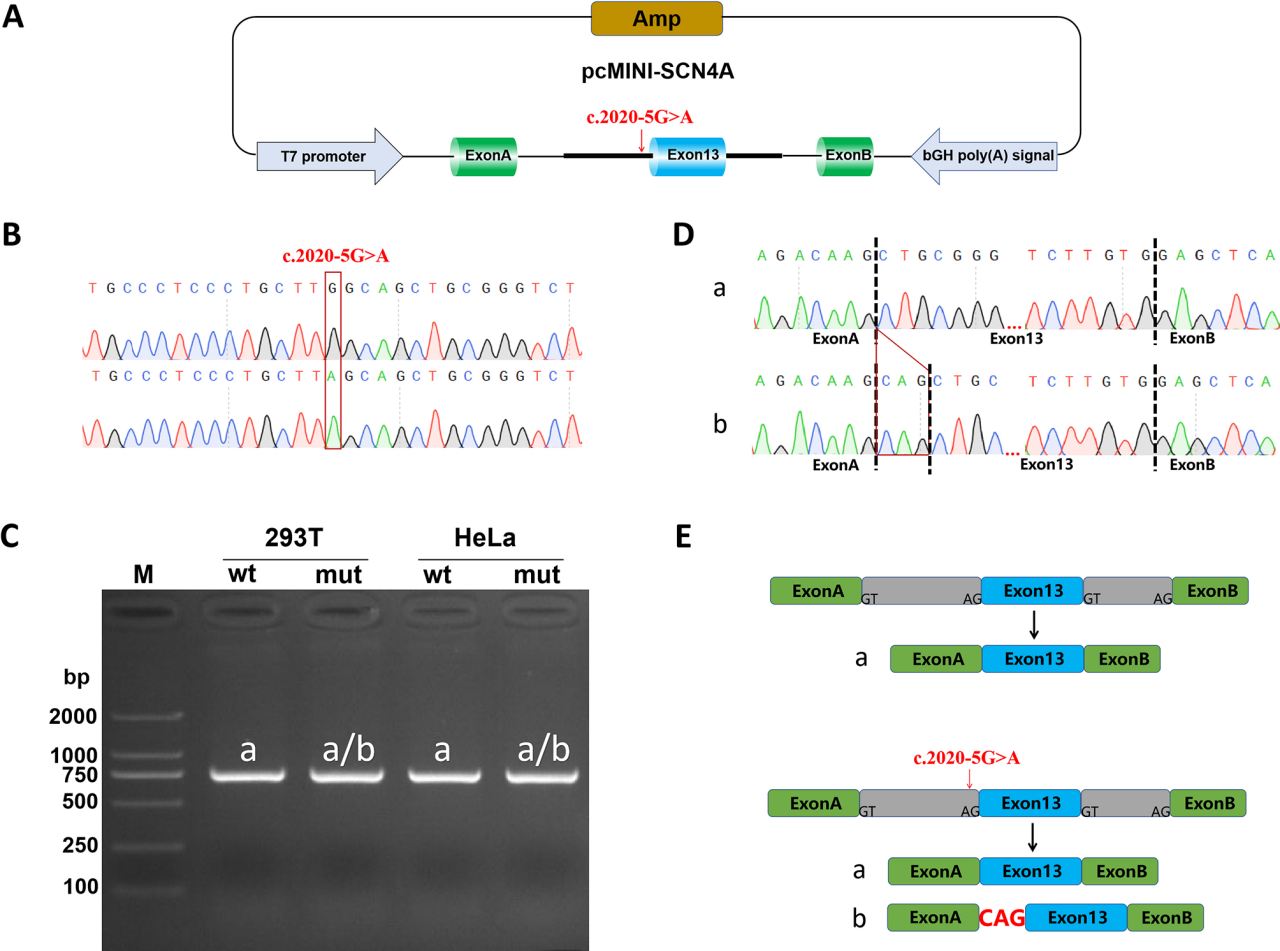
The effect of variant c.2020-5G > A of *SCN4A* on mRNA splicing. **(A)** A Schematic diagram of the vector constructs; **(B)** Sequencing results of minigene constructs, UP: wildtype, down: mutation; **(C)** agarose gel electropherograms after RT-PCR; The bands in 293T and HeLa cells are labeled as a, b; Band a represents the Wild-type fragment with 746bps (normal splicing), band b represents the abnormal splicing fragment with 749bps. Band b was 3 bp larger than band a, indistinguishable after electrophoresis on 1% agarose gel; **(D)** the sequencing results of the bands after TA cloning correspond to the minigenes; **(E)** Schematic representation of minigene splicing. Band b is an abnormal band with 3 bp retention to the right of Intron12 and Exon A -▽Intron12 (3 bp) -Exon13-Exon B (749 bps)

The minigene pcMINI-SCN4A-WT/MT vectors were constructed to explore the splicing effect of this variant (Fig. 3A and B). Although RT-PCR analysis showed a single band with the expected size of 746 bps for both WT and MT minigenes in two different cell lines (Fig. 3C), the mutant products showed overlapping peaks in Sanger sequencing (data not shown). TA cloning was followed by Sanger sequencing with two bands, naming the small band as a and the large band as b (Fig. 3D). The sequencing results showed that band a was a normal band containing Exon A-Exon13-Exon B (746 bps), while band b was an abnormal band retaining 3 bp to the right of Intron12 and Exon A -▽Intron12 (3 bp) -Exon13-Exon B (749 bps) (Fig. 3E).

The results of the in vitro minigene assay showed that the mutation c.2020-5G > A affects the normal splicing of the *SCN4A* mRNA. The retention of 3 bp of Intron12 on the cDNA and protein levels resulted in the variant c.2019_2020insCAG (p.Leu673_Leu674insGln), and 3 bp on the right side of Intron12 did not cause subsequent read frame change, producing a protein with a length of 1837 aa.

## Discussion

PPP is a genetic disorders characterized by episodes of muscle weakness caused by mutations in genes encoding channel proteins of skeletal muscle [7]. The molecular diagnosis of PPP was previously based on hot spots, exon-by-exon screening of the reported genes or targeted panel sequencing [13]. The latest and large cohort studies on PPP are mainly concentrated in patients from the United States and Europe, and have identified a heterozygous pathogenic mutation in 60–70% of patients meeting clinical criteria [11, 12]. We performed genetic analysis for 37 Chinese patients using a gene panel and WES, which elucidated genetic causes in 59.5% (22/37) of patients in our cohort. This was consistent with another study in the Chinese population, identifying candidate gene variants in 65.0% (39/60) of patients [13].

In the United States and Europe, the most commonly affected gene in patients with HypoPP was *CACNA1S*, followed by *SCN4A* [2]. In up to 3% of patients, some mutations have been found to be associated with thyrotoxic HypoPP, most commonly affecting *KCNJ18* and *KCNE3* [19, 20]. It has been reported that very few patients have been found to carry *RYR1* gene variants [21]. In addition to covering the *CACNA1S* and *SCN4A* genes, our panel included five additional genes associated with periodic paralysis, such as *RYR1, KCNE3, KCNJ18, KCNJ2* and *KCNJ5*. Among 21 patients with HypoPP, 15 were classified as having HypoPP-2 with *SCN4A* variants, and 6 as having HypoPP-1 with *CACNA1S* variants. Based on these results, we speculated that the *SCN4A* gene may be the most common causal gene in patients with HypoPP in China, different from Europe and the United States. This result was consistent with another study in the Chinese population, indicating that *SCN4A* accounts for the majority of HypoPP, while *CACNA1S* mutations are relatively rare [13]. Brugnoni et al. found that among 51 patients with HypoPP, 4 Asian patients were found to carry pathogenic variants of the *SCN4A* gene [9]. This may imply that *SCN4A* is the most commonly affected gene in Chinese or Asian patients with HypoPP.

In our cohort, only one of the 22 probands was female, and the penetrance was 95.5% (42/44) in males, compared to 73.4% (14/19) in females. These results are consistent with previous literature reports that the penetrance of periodic paralysis was lower in women than in men, which may be associated with sex hormones [4].

**Fig. 4:**
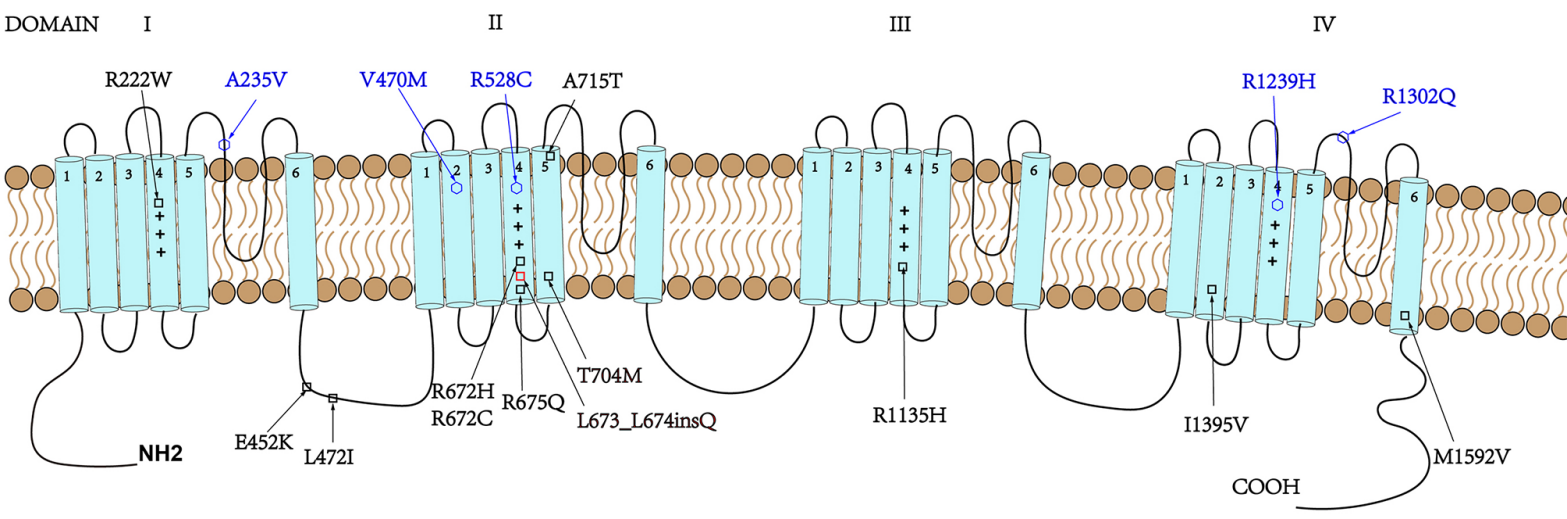
*CACNA1S* and *SCN4A* mutations included in our study are pictured in the same structural model channel. Both Cav1.1 and Nav1.4 channels are composed of four homologous transmembrane domains (DI–DIV); each containing six transmembrane domains (S1-S6). Plus (+) symbolizes the positive charges in segment 4 of the calcium/sodium channels. Black square: mutations in the Nav1.4 channel; Blue hexagon: mutations in the Cav1.1 channel. The figure is adapted from Brugnoni et al. (2022)

**Fig. 5:**
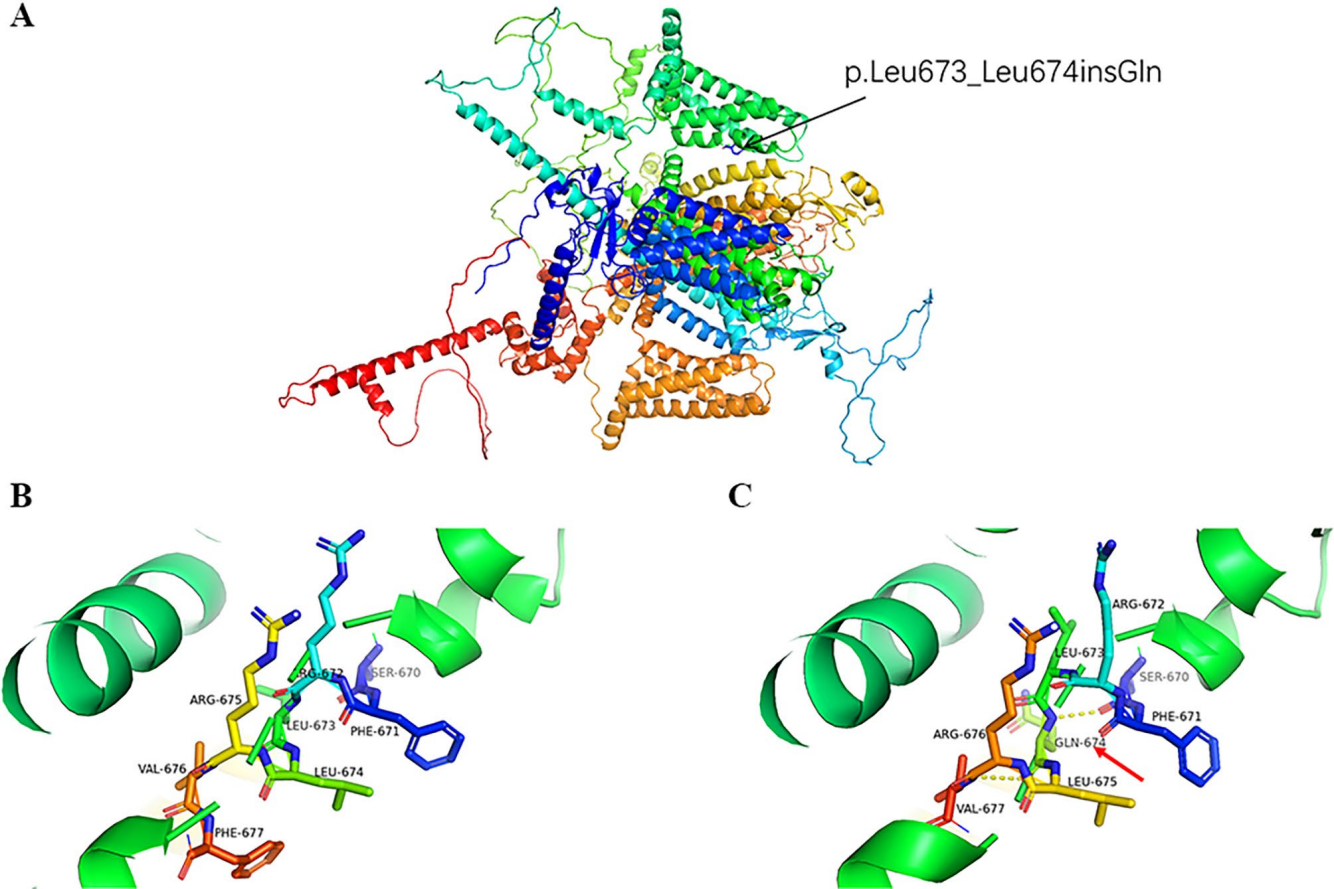
The 3D molecular structure of *SCN4A*. **(A)** Homology model of *SCN4A* mutant protein generated using the SwissModel online server. Magnified views of the wild-type **(B)** and mutant Leu673_Leu674insGln **(C)** are shown respectively. The H-bonds are shown as yellow dashed lines, and Gln is indicated by a red arrow

The α subunits of the skeletal muscle Cav 1.1 channel and Nav1.4 channels are composed of four homologous transmembrane domains (DI-DIV). Within every domain, six transmembrane helical segments are present (S1-S6) consisting of two distinct modules: the voltage-sensor domain (S1-S4) and the pore domain (S5-S6) (Fig. 4). The S4 segment of each homologous domain contains repeating motifs of one positively charged amino acid followed by two hydrophobic residues (R/K-X-X; X-hydrophobic residues). The outward movement and rotation of the S4 helices in response to depolarization inducse a conformational change within the channel, which opens the main ion-conducting pore [22, 23]. Most of the *CACNA1S* and *SCN4A* mutations identified so far affect residues located in segment S4 of all four domains of the protein, which represents the voltage-sensing apparatus of the channel and leads to the replacement of a highly conserved positively charged arginine with a weakly charged or neutral amino acid residue [22, 24]. It has been reported that the common mutational hotspots in the *CACNA1S* gene include p.R174W (DI-S4), p.R528G/C/H (DII-S4), p.V876E (DIII-S3), p.R897S (DIII-S4), p.R900G/S (DIII-S4), p.R1086H (cytoplasmic), p.R1242G/S (DIV-S4), and p.R1239H/G (DIV-S4) [8, 25–30]. Similarly, the hotspots in the *SCN4A* gene include p.R222W (DI-S4), p.R669H (DII-S4), p.R672S/G/C/H(DII-S4), p.N820Y (cytoplasmic), p.R1129Q (DIII-S4), p.R1132Q (DIII-S4), p.R1135H/C (DIII-S4), and p.P1158S (Ex) [12, 31–36]. More than half of the variants (13/22) are located in the S4 segment of four domains and result in the replacement of an arginine in our study cohort (Supplement Table 1; Fig. 4). Our results indicate that the variants p.R675Q (DII-S4) and c.2020-5G > A may also be relatively common mutational hotpots in Chinese HypoPP-2 patients. The splicing variant c.2020-5G > A contributed to a non-frame-shift change (p.Leu673_Leu674insGln) in segment S4 of DII. The insertion of the hydrophilic amino acid Gln may disrupt the repeating motif of the S4 segment, stabilizing the hydrogen bonding interactions fo the Gln with Ser670 and Val677 (Fig. 5), eventually leading to a conformational change of the S4 helices, affecting the voltage-sensing apparatus. This variant was classified as “likely pathogenic” according to guidelines determined by the ACMG (PS4 + PM2 + PP3).

Initially, we started to analyze PPP patients using panel sequencing to identify pathological mutations in known genes. With the development of high-throughput sequencing technologies, we more recently began using WES to analyze the underlying genetic variations. Indeed, WES could be useful for the detection of mutations in regions or genes not included in our panel, as well as macro deletions or duplications. However, WES was not able to identify any mutation in HypoPP patients already screened for mutations using the panel. Moreover, the overall detection rate was comparable between the panel (54.5%) and WES (61.5%). To our best knowledge, this is the first study that analyzes the diagnostic utility of WES in patients affected by PPP, and the results do not support its superiority in the molecular diagnostic process of these diseases compared to the panel.

Mutations of the *SCN4A* are associated with various neuromuscular disorders, including HyperPP, HypoPP2, paramyotonia congenita and congenital myasthenic syndrome et al. [37–41]. *SCN4A* mutations leading to periodic paralysis or nondystrophic myotonia have been found located throughout every domain and segment of this channel [42]. It has been reported that similar or even the same mutations in the *SCN4A* gene can cause distinct clinical disorders [43]. For example, the variant R675Q can cause HyperPP, HypoPP-2 as well as NormoPP [42, 44–46]. In our study, the variant R675Q was the most common mutation site and was associated with HypoPP-2 in four families. Phenotypic differences between individuals harboring the same *SCN4A* mutation indicate that the genetic background and other factors perhaps contributed to the clinical expression of some variants.

HyperPP is characterized by attacks of flaccid limb weakness, hyperkalemia (serum potassium concentration > 5 mmol/L) and/or provoking/worsening of an attack by oral potassium intake, and normal serum potassium between attacks. In approximately half of the individuals, the onset is in the first decade of life, and the prevalence of HyperPP is approximately 0.17/100,000–0.06/100,000 [47]. There was only one HyperPP case (case 5) in the cohort, and included multiple patients in the family. The onset of the proband was 1 year old, and other patients were between 3 and 9 years old in the same family. The age at the appearance of the most severe symptoms was between 10 and 25 years. The relatively earlier onset time in patients with HyperPP than HypoPP in our cohort is consistent with previous reports that symptom onset is typically in the second decade for HypoPP while HyperPP tends to present earlier in childhood [11]. Mutations of *SCN4A* that are associated with HyperPP affect the gating behavior of this channel and produce gain-of-function defects characterized by impaired inactivation and/or enhancement of activation [48, 49]. We identified a heterozygous c.2111 C > T (p.Thr704Met) variant of *SCN4A* in case 5, one of the most common HyperPP mutations causing disrupted slow inactivation [50].

Patients with NormoPP often develop symptoms around the age of 10, generally in the form of paroxysmal muscle weakness with normal serum K^+^ concentrations. However, sometimes it was difficult to determine whether a normokalemic variant was a distinct entity or whether it was simply a form of HyperPP. One may see misleading measurement results of the serum potassium levels, when it is made too far apart from the progressive onset of the attack [21]. In a series of patients carrying the common T704M *SCN4A* variant, known to cause HyperPP, only 50% were shown to have hyperkalemia during attacks [49], and the diagnostic category of NormoPP was thought to be doubtful [51]. In addition, NormoPP is the rarest subtype of PPP and a further limitation of our study is the relatively small sample size. Therefore, it is understandable that no cases of NormoPP were found in our study.

In this cohort, 2 variants of *SCN4A* and 2 variants of *CACNA1S* were classified to uncertain significance according to ACMG. The Novel variant p.Ile1395Val was located in the DIV-S2 and the adjacent variant p.Ile1393Thr has been reported to be associated with paramyotonia congenita [52]. Another novel variant of *SCN4A*, p.Leu472Ile was located in the cytoplasmic Domain I. The remaining 2 variants in *CACNA1S*, p.V470M and p.Arg1302Gln, are distributed in DII-S2 and the S5-S6 loop of extracellular Domain 4, which are pore-forming domains. Although they are rare variants and might be pathogenic, a detailed co-segregation analysis and functional studies are still required to validate these results.

## Conclusion

Our results suggest that *SCN4A* alleles are the overwhelming cause of PPP in our cohort, with the remainder caused by *CACNA1S* alleles, which conversely are the predominant cause in Europe and the United States. This study identified 3 novel *SCN4A* and 2 novel *CACNA1S* variants. There was no further benefit of WES over the implementation of complete sequencing of targeted genes included in our panel in clinically defined HypoPP. As HypoPP is a rare disease, there are understandable difficulties related to the collection of data, and further studies on large cohorts of patients are needed to clarify our findings.

### Electronic supplementary material

Below is the link to the electronic supplementary material.

Supplementary Material 1

## Data Availability

All data generated or analyzed during this study are included in this published article.

## Abbreviations

PPP: Primary periodic paralysis
WES: Whole exon sequencing
