# Supplementary material for: Genetic analysis of 37 cases with primary periodic paralysis in Chinese patients
Source: Orphanet J Rare Dis. 2024 Apr 12;19:160. doi: 10.1186/s13023-024-03170-5 (PMC11015673; doi:10.1186/s13023-024-03170-5)
Supplement: Supplementary file 1 — Supplementary Material 1 [file 13023_2024_3170_MOESM1_ESM.docx]

Supplement table 1. Distribution of mutations in the 22 mutated PPP patients

| gene | Mutation | Patients | exon | Structural position |
| --- | --- | --- | --- | --- |
| SCN4A | c.2024G>A(p.Arg675Gln) | 4 | 13 | DII-S4 |
|  | c.2020-5G>A | 2 |  |  |
|  | c.2111C>T (p.Thr704Met) | 1 |  | DII-S5 |
|  | c.2143G>A(p.Ala715Thr) | 1 |  |  |
|  | c.2015G>A(p.Arg672His) | 1 | 12 | DII-S4 |
|  | c.2014C>T(p.Arg672Cys) | 1 |  |  |
|  | c.1414C>A(p.Leu472Ile) | 1 | 9 | cyto |
|  | c.1354G>A(p.Glu452Lys) | 1 |  | cyto |
|  | c.664C>T(p.Arg222Trp) | 1 | 5 | DI-S4 |
|  | c.4183A>G(p.Ile1395Val) | 1 | 23 | DIV-S2 |
|  | c.4774A>G(p.Met1592Val) | 1 | 24 | DIV-S6 |
|  | c.3404G>A(p.R1135H) | 1 | 18 | DIII-S4 |
| CACNA1S | c.3716G>A(p.Arg1239His) | 2 | 30 | DIV-S4 |
|  | c.1408G>A(p.V470M) | 1 | 11 | DII-S2 |
|  | c.1582C>T(p.R528C) | 1 |  | DII-S4 |
|  | c.704C>T(p.Ala235Val) | 1 | 6 | EX |
|  | c.3905G>A(p.Arg1302Gln) | 1 | 32 | EX |
